# Recombinant Expression and Biophysical Characterization of a Druggable *Schistosoma mansoni* Universal Stress G4LZI3 Protein

**DOI:** 10.34172/apb.2022.035

**Published:** 2021-01-31

**Authors:** Abiola Fatimah Adenowo, Priscilla Masamba, Ndibonani Kebonang Qokoyi, Babatunji Emmanuel Oyinloye, Abidemi Paul Kappo

**Affiliations:** ^1^Department of Medical Biochemistry, Faculty of Basic Medical Sciences, Lagos State University College of Medicine, PMB 21266, Ikeja, Lagos, Nigeria.; ^2^Molecular Biophysics and Structural Biology (MBSB) Group, Department of Biochemistry, University of Johannesburg, Kingsway Campus, Auckland Park 2006, South Africa.; ^3^Department of Biochemistry, Afe Babalola University, PMB 5454, Ado-Ekiti 360001, Nigeria.

**Keywords:** Biophysical characterization, G4LZI3, Recombinant proteins, Schistosoma mansoni, Schistosomiasis

## Abstract

***Purpose:*** Universal stress protein (USP) from *Schistosoma mansoni*, designated as G4LZI3, waspreviously hypothesised as a druggable target and vaccine candidate for human schistosomiasis.The purpose of this study is to characterize a purified recombinant G4LZI3 preliminarily forsubsequent structural characterization, which will provide baseline structural data for futurefunctional studies for the discovery, design and development of new schistosomal drugs for thetreatment, control and elimination of schistosomiasis.

***Methods:*** Restriction digest analysis of a GenScript-synthesised codon-optimised G4LZI3gene construct was carried out to ascertain its integrity and size. Thereafter, the pQE30-G4LZI3 construct was transformed into an M15 bacterial expression host. Transformed cellswere induced with isopropyl β-D-thiogalactoside for recombinant protein expression of anappreciable amount of pQE30-G4LZI3, which was subsequently purified with fast proteinliquid chromatography (FPLC) and a size exclusion chromatographic purification scheme.Preliminary biophysical characterization of the 6X His-tagged G4LZI3 was done to determineits secondary structure characteristics and protein stability.

***Results:*** A molecular weight protein of 20.3 kDa was confirmed subsequent to restriction digestanalysis, while heterologous protein expression yielded a highly soluble and considerableamount of histidine-tagged G4LZI3 protein, which was successfully purified to homogeneity.Biophysical characterization indicated that the protein was well folded, heat-stable, had thefunctional groups and secondary structure composition required and was thus amenable tofurther structural characterization and determination.

***Conclusion:*** Biophysical characterization of purified G4LZI3 showed that further structuralstudies can be embarked upon on the use of G4LZI3 as a druggable target and possibly avaccine target against schistosomiasis via vaccinomics.

## Introduction

Human schistosomiasis persists as one of the neglected tropical diseases, with over 261 million individuals infected worldwide; a great proportion of these are located in Africa, South America and Asia. It is second to malaria in terms of prevalence and is the second top neglected tropical disease after hookworm infection in sub-Saharan Africa.^1,2^ This debilitating parasitic disease is caused by the trematode-worms of the genus *Schistosoma.* Three members of this class are known to infect humans: *Schistosoma mansoni* and *Schistosoma japonicum* are responsible for intestinal schistosomiasis, while *Schistosoma haematobium* causes urinary schistosomiasis.^2,3^ The lifecycle of the blood-dwelling flatworms involves two developmental phases, a sexual phase in the definitive human host and an asexual phase in the freshwater snail host.^4,5^ Schistosomiasis occurs owing to immunological reactions caused by the *Schistosoma*eggs entrapped in tissues. Various antigens released from the eggs trigger a granulomatous reaction that involves T-cells, eosinophils and macrophages, resulting in clinical infections.^5,6^ The symptoms and signs resulting from infection depend on the location and number of eggs trapped within the affected tissues. At the onset, the inflammatory reaction is reversible but in the later phases of the disease, the pathology is linked with collagen deposition and fibrosis, which result in irreversible organ damage and chronic disability.^7,8^ Various strategies have been employed to control the spread of the disease. These include health education and improved sanitation, use of biological control approaches to eliminate the intermediate snail host of the parasites and mass administration of praziquantel (PZQ).^2,9^ So far, PZQ, a pyrazinoisoquinole anthelmintic, is the only frontline drug for the treatment of all forms of schistosomiasis. However, this drug has several limitations, including its ineffectiveness against the various developmental stages of the worm, inability to kill immature worms at early-stage infection and reports of drug resistance in various endemic communities. On the basis of these limitations and shortcomings of PZQ, there is a dire need for concerted efforts to develop alternative drugs for the treatment of human schistosomiasis, as well as the formulation of a vaccine against the disease.^2,4,5^

The genome of *Schistosoma* is known to encode various proteins that have the universal stress protein (USP) domain (Pfam Accession number: PF00582). USPs have been discovered in yeast, archaea, bacteria, fungi and plants. The expression of this stress protein is typically prompted by numerous environmental stressors such as life-threatening temperature, noxious chemicals, malnourishment, oxidants, heavy metals, electron transport chain uncouplers, acids and antibiotics.^4,10,11^ Several recombinant proteins produced from microorganisms and higher organisms are being used as therapeutic agents and vaccine candidates after undergoing biophysical and structural characterization. Thus, a USP, G4LZI3, from *S. mansoni* has been proposed as a useful therapeutic intervention against schistosomal infections.^4,5,10^ Since the USP has been identified as a possible therapeutic agent against human schistosomiasis, this study seeks to produce recombinant *S. mansoni* USP G4LZI3 via heterologous protein expression, purify the expressed protein to remove undesired proteins produced during recombinant expression as well as other impurities and consequently provide preliminary biophysical data to enable its utilisation in the discovery, design and development of new drugs against schistosomiasis.

## Materials and Methods

### Restriction digest analysis of synthesised G4LZI3 gene construct

Using the amino acid sequence of USP available on the NCBI database, the DNA sequence comprising the coding region of *S. mansoni* G4LZI3(XP_018646651.1) was chemically synthesised by GenScript Inc (GenScript Corp, Piscataway, NJ, USA). A restriction digest analysis was carried out according to the previously described method with modifications,^12,13^ to ascertain the integrity and size of the synthesised gene construct. The reaction set-up consisted of 1 μL of BamH1, 1 μL of Hind III, 1 μL of pQE30-G4LZI3 (1 μg/μL), 2 μL restriction enzyme buffer (0.01 M Tris/HCl pH 7.4, 0.001M EDTA pH 8.0) and 15 μL of deionised water. The reagents were mixed in a vial and incubated in a water-bath at 37°C for 40 minutes. Afterwards, electrophoresis using a 1.2% agarose gel (prepared by adding 1.2 g of agarose gel to 120 mL 1X TAE buffer and 10 μL ethidium bromide) electrophoresis was done to confirm the integrity of the plasmid construct.

### Bacterial transformation, heterologous expression and purification of pQE30-G4LZI3 construct

Bacterial transformation and heterologous expression were done using previously described methods with modifications.^13,14^ The confirmed pQE30-G4LZI3 plasmid DNA was mixed with 100 µL pre-thawed competent M15 *Escherichia coli* cells (Qiagen, Hilden, Germany) in a tube and was incubated on ice for 20 minutes and then heat-shocked at 42°C for 45 seconds. Further incubation was done on ice for 5 minutes; afterwards, 900 µL of pre-warmed Luria Bertani (LB) medium with ampicillin was added to the suspension before incubation at 37°C for an hour. Subsequently, 50 µL of the transformed cells was spread out on LB agar plates containing 100 µg/mL ampicillin and the plates were incubated overnight at 37°C. The next morning, four single colonies were randomly picked and inoculated into four tubes containing 5 mL of 2XYT medium with 100 µg/mL ampicillin. The culture tubes were incubated at 37°C for 4 hours, with vigorous shaking. Subsequent to incubation, 1 mL culture from each culture tube was put into eight clean sterile tubes. Four tubes were labelled un-induced control culture and then the other four tubes were labelled induced culture, after the addition of 0.5 mM isopropyl β-D-thiogalactoside [IPTG] (Sigma, St Louis, MO, USA). All culture tubes were incubated for an additional 2 hours at 37°C with vigorous shaking, followed by centrifugation at 6000 × *g* for 5 minutes in order to harvest the cell pellets. The harvested pellets from both the induced and un-induced sets of tubes were re-suspended in 50 µL PBS and 20 µL from all cell suspensions was electrophoresed on a 16% SDS-PAGE gel (Bio-Rad, Neuberg, Germany).

After expression screening, 100 µL of the positive clones was inoculated into 100 mL of 2XYT medium broth (Peptone 1.6%, Yeast 1%, NaCl 0.5%) containing 100 µg/mL of ampicillin. The culture flask was allowed to shake briskly till the next morning at 37°C. The following morning, the culture was made up to 2000 mL with 2XYT containing 100 µg/mL of ampicillin and incubated, with shaking, at 37°C. Thereafter, 0.5 mM of IPTG (Sigma, St Louis, MO, USA) was added at OD_600_ to induce protein expression and the culture was further incubated at 25°C with vigorous shaking till the following morning. The total bacterial lysates were harvested by centrifugation for 40 minutes at a temperature of 4°C at 6000 × *g*and were thereafter stored at -80°C.

### Extraction and purification of G4LZI3 with fast protein liquid chromatography

Total bacterial lysates stored at -80°C were thawed on ice, followed by the addition of a protease inhibitor cocktail into the sample to prevent degradation. Sonication of the sample at 15-second intervals for 4 minutes was done to allow for the release of the protein from the cells. The bacterial lysates were then subjected to centrifugation at 20 000 × g for 30 minutes at 4°C to obtain a clear lysate, which was then filtered using a 0.45-micron filter to remove any contaminating particles in the sample prior to affinity purification by the AKTA explorer (GE Healthcare, Wisconsin, USA). Subsequent to the extraction of the bacterial lysate, protein purification was done, using the previously described protocol with some modification.^15^ To begin with, the AKTA fast protein liquid chromatography (FPLC) purifier system containing Ni-Sepharose beads (GE Healthcare, Wisconsin, USA) was washed with five column volumes (CV) of deionised water. This was then followed by 30 CV of equilibration where line A was put into the equilibration buffer and line B in the elution buffer. This was done to displace all the water out of the lines. Thereafter the column was equilibrated with 1 CV of equilibration buffer (50 mM Tris at pH 8, 40 mM imidazole, 500 mM NaCl). The superloop was then connected and 50 mL of the sample was loaded into the superloop. The sample was then injected into the column and eluted with 20 CV of elution buffer (50mM Tris at pH 8, 300mM imidazole, 500mM NaCl). At this stage, 5 mL fractions of elute were collected. The column was then washed with 5 CV of equilibration buffer to get rid of any unbound proteins in the column. Thereafter, an SDS PAGE gel was run from the collected fractions in reference to the peaks seen on the chromatogram that was drawn on a connected computer as the sample was running in the column.

### Purification of G4LZI3 by size exclusion chromatography

Thereafter, size exclusion chromatography (SEC) was done as a second purification step using the method initially described, with some adjustments.^16^ Fractions from affinity purification by the AKTA, containing the G4LZI3 protein, were pooled and concentrated down to a final volume of 2 mL using a 3K Macrosep^®^ Advance Centrifugal device (Pall Corporation, Port Washington, New York) and centrifuged at 4000 ×g using a 4440 rotor for 20 minutes. The concentrated sample was then filtered using a 0.22-micron filter and a syringe prior to loading it into a Superdex 75 (S75) prep grade (pg) column of size 16 mm in diameter and 600 mm bed height (16/600) (GE Healthcare, Wisconsin, USA). Prior to the introduction of the sample, column calibration was done using Conalbumin (75 kDa), Carbonic anhydrase (29 kDa) and Ovalbumin (44 kDa) as standards. Thereafter, the column was equilibrated with 150 mM NaCl and 50 mM Tris pH 8.0. The sample was then injected into the S75 column running at a flow rate of 0.5 mL/minute. At this stage a chromatogram was drawn on a connected computer, while 5 mL fractions were collected in the fraction collector and used to analyse the data on a 15% SDS PAGE gel. Fractions containing the G4LZI3 protein were thereafter pooled and concentrated down further to a final volume of 1 mL using 3K Macrosep^®^ centrifugal units (Pall Corporation, Port Washington, New York). The purified protein sample was then snap-frozen and kept at -80°C.

### Biophysical characterization of G4LZI3

#### Fourier transform infrared spectrum of G4LZI3

The Fourier transform infrared (FTIR) spectrum were collected using the protocol described earlier with modifications.^17^ It was done with 40 µL of diluted protein sample on a Perkin–Elmer 1725 series FTIR spectrophotometer^17^ (Perkin–Elmer Corporation, Connecticut, USA) that has a deuterated triglycine sulphate detector. The spectrophotometer was attached to a Perkin–Elmer model 7300 computer with infrared data system (IRDM) software. The sample was positioned in close contact with the attenuated total reflection element (ZnSe crystal, 45 ends) at 37°C. FTIR data were acquired over the region 4000–650 cm^-1^ by running 32 scans at a resolution of 4 cm^-1^ with apodisation. The spectrum was collected as absorbance values at each data point.

### Dynamic scanning calorimetry

A Perkin-Elmer DSC 6000 (Perkin–Elmer Corporation, Connecticut, USA) instrument was used to investigate thermal stability and structural conformation of G4LZI3. The lyophilised G4LZI3 protein was dissolved in distilled water and filtered. Thereafter, the PBS reference solution and G4LZI3 solution were degassed by constant stirring and were then put into the reference cells and sample cells respectively with an air-tight syringe and subjected to heating at 20°C to 150°C at a rate of 10°C/min. Then, the differential power compensation required to keep the sample cell and reference cell at an equal temperature was documented. The value obtained was used for working out the calorimetric enthalpy and mid-point transition temperature (Tm) required for determining the thermostability of the protein. The data acquired were interpreted using Origin software (MicroCal Software, Inc., MA, USA).^18^

### Fluorescence spectrum of G4LZI3

A Perkin Elmer fluorescence spectrophotometer (Perkin–Elmer Corporation, Connecticut, USA) instrument and 0.5 cm × 0.5 cm quartz cuvettes were used to obtain fluorescence spectra of the recombinant G4LZI3 protein based on the method described earlier, with modifications.^19^ The spectra were documented at a protein concentration of 0.10 mg/mL in 50 mM Tris-HCl buffer (pH 7.0) at room temperature. Tris-HCl buffer was used as blank, while the excitation wavelength was 270 nm. The intrinsic fluorescence emission scanning was documented within the wavelength of 200-450 nm.

## Results

### Restriction digest analysis and heterologous expression of G4LZI3 gene

**Figure 1:**
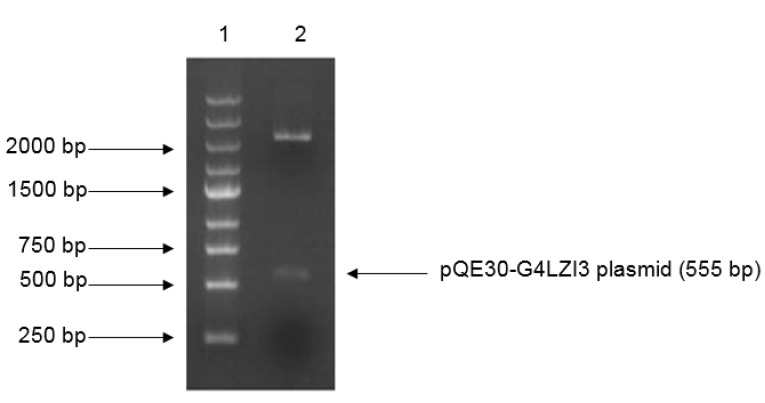
**Restriction digest gel for confirmation of G4LZI3 insert fragment.** Lane 1 is the DNA marker, lane 2 is the plasmid digested by BamH1 and HindIII.

**Figure 2:**
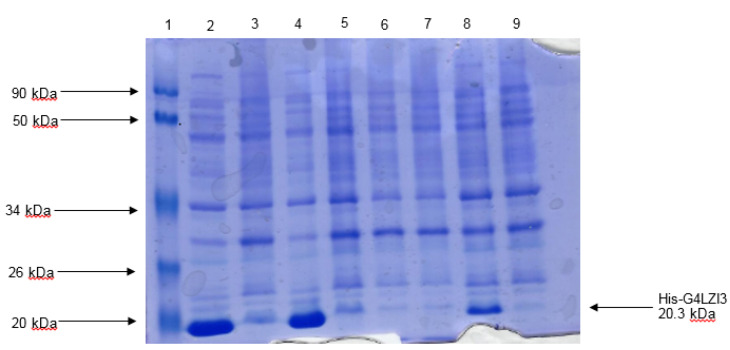
** Expression screen of transformed colonies from pQE30-G4LZI3 for protein expression.** Lane 1 is the protein molecular weight marker, lanes 2, 4, 6 and 8 indicate the total bacterial cell lysates after induction of protein expression with addition of 0.5 mM IPTG, while lanes 3, 5, 7 and 9 indicate the corresponding total bacterial cell lysates of the un-induced cultures. The induced lanes (2, 4 and 8) show bands of approximately 20.3 kDa, corresponding to the His-G4LZI3 fusion protein.

Restriction digest analysis confirmed the integrity and size (555 bp) of the construct, as shown in the 1.2% agarose gel elecrophoresis in Figure 1. The SDS-PAGE analysis gel presented in Figure 2 shows the expression screening of total bacterial lysates of indiscriminately selected bacterial colonies from the transformation plate. Presented in the SDS-PAGE gel are lanes 2-9, consisting of alternating pairs of induced and un-induced culture samples; the conspicuous protein bands are seen in the induced culture lanes but are lacking in the un-induced culture lanes. It is noteworthy that 6X His-G4LZI3 protein is well expressed, with a molecular weight of approximately 20.3 kDa, as seen in the conspicuous protein bands in the induced culture lanes (Figure 2)

### Affinity purification of G4LZI3

**Figure 3:**
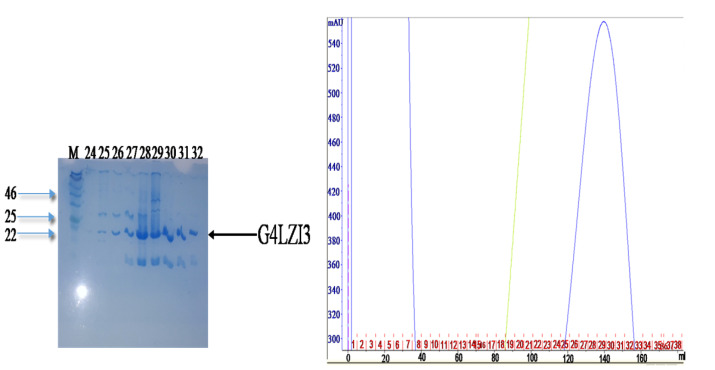
**
Affinity purification chromatogram and SDS PAGE gel.** The peak was observed at a high imidazole concentration, indicating elution of G4LZI3 protein. The SDS PAGE gel on the left side shows fractions from the peak. The tight bands at approximately 20.3 kDa indicate partially successful purification of the protein. The bands observed at around 20 kDa are indicative of either protein degradation or the presence of a contaminating protein, hence a second purification step was necessary.

Immobilised metal affinity chromatography (IMAC) using the GE Healthcare AKTA FPLC (Wisconsin, USA) purifier system was used as an initial purifying step in this study. The high imidazole concentration competed with the histidine tag, hence the subsequent release of the bound protein from the beads. In the affinity chromatogram of the 6XHis-G4LZI3 (Figure 3), the green line represents imidazole, which is responsible for the elution of the protein. A peak was observed at a high imidazole concentration and was indicative of eluted G4LZI3 protein from the column. Fractions from the peak (fraction number 24, 25, 26, 27, 28, 29, 30, 31 and 32) were collected and run on a 15% SDS PAGE gel for analysis. The SDS PAGE gel shows tight bands at approximately 20.3 kDa, indicating successful first-step purification of the protein. However, similar bands were observed below 20 kDa. This is more likely to be an indication of protein degradation but may also be a contaminating protein, hence a second purification step was necessary.

### Size exclusion chromatography

**Figure 4:**
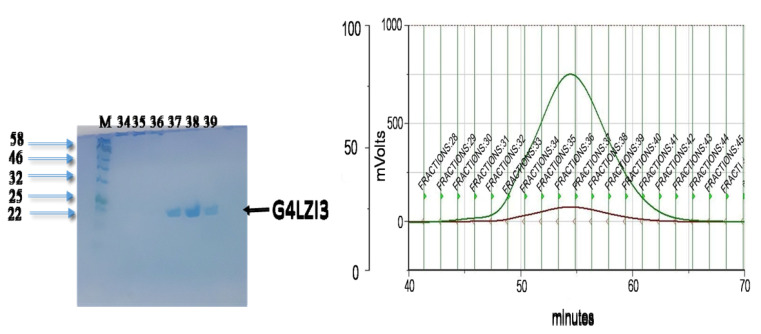
**
SEC chromatogram and SDS PAGE gel.** Chromatogram showing separation of G4LZI3 from high molecular weight impurities present in the affinity-purified sample. SDS PAGE gel on left shows G4LZI3 protein to be running at the expected size of 20.3 kDa from fractions 34, 35, 36, 37, 38 and 39.

To purify the G4LZI3 protein to homogeneity, SEC was done as the second purification step using the Superdex75 PG column of size 16 mm in diameter and 600 mm bed height (16/600) (GE Healthcare Sciences). The gel filtration chromatogram in Figure 4 shows the separation of the G4LZI3 from high molecular weight impurities present in the affinity-purified sample. The retention time, prior to elution of about 70 minutes, showed that the protein was small enough to enter the pores of the stationary phase and hence allowed for the purification of the protein. To assess the extent of purity, as well as to confirm which peak corresponded to the eluted protein, fractions were collected from the SEC chromatogram and run on an SDS PAGE gel electrophoresis for analysis; this is shown in the SDS PAGE gel in Figure 4. The SDS PAGE gel showed G4LZI3 protein to be running at the expected size of 20.3 kDa from fractions collected in the G4LZI3 peak (fractions 34, 35, 36, 37, 38 and 39). Therefore, it can be concluded that the G4LZI3 protein was successfully purified to homogeneity by SEC.

### Fourier transform infrared spectroscopy analysis of His-G4LZI3 protein

**Figure 5:**
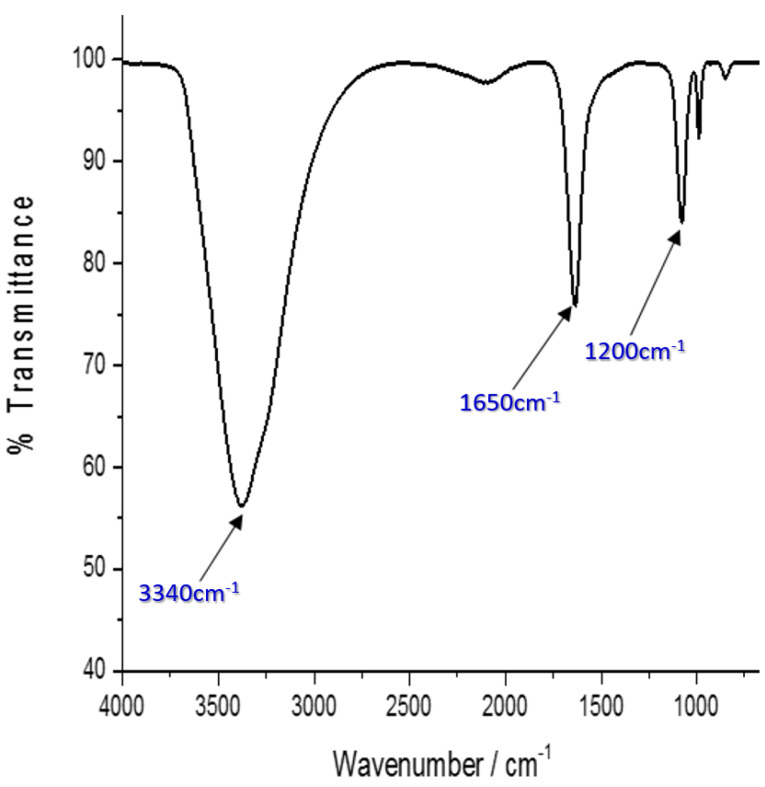
FTIR spectrum of G4LZI3. Three prominent peaks are shown, indicating the secondary structure element and functional groups present in the protein.

The presence of secondary structure elements, as well as functional groups in the His-G4LZI3 protein, was investigated using FTIR. Figure 5 indicates the presence of three noticeable peaks; the peak corresponding to 3 340 cm^-1^ signifies the amide A group and it arises owing to the resonance between the first overtone of amide II and the N-H stretching vibration. The second, corresponding to approximately 1 650 cm^-1^ may be accredited to the amide I band. This peak is linked to the vibrational stretching of the C = O bond, hence the position and shape of the amide I band are used for the study of protein secondary structure.^20^ It is proposed that the sharp amide I band at 1 650 cm^-1^ is indicative of an α-helix, which is the major element in the G4LZI3 protein secondary structure. Alongside this is a third peak, which is at about 1 200 cm^-1^ and represents the secondary amide III band that is produced by the C = N stretching vibration coupled with the N-H plane bending vibration.^21^

### Thermal stability of G4LZI3

**Figure 6:**
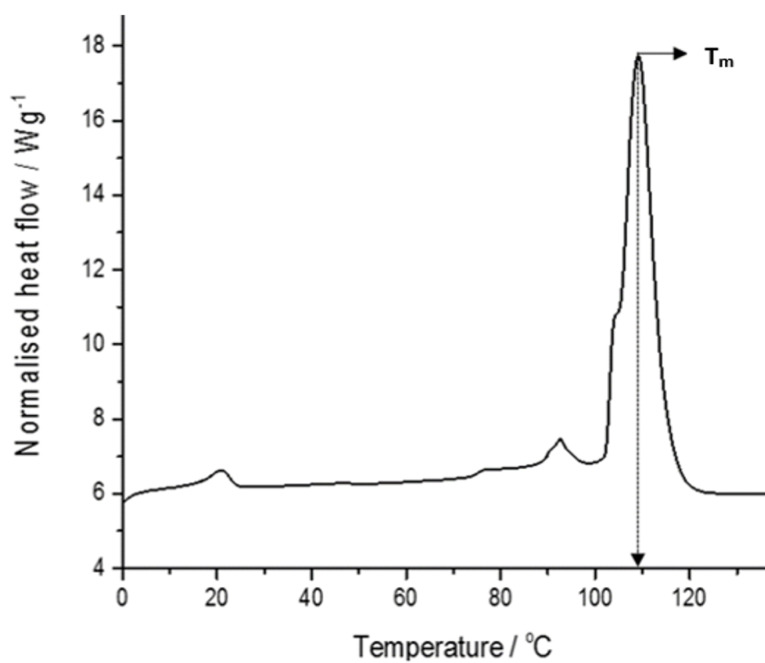
**
A DSC thermogram of G4LZI3.** The protein is shown to have a transition temperature of approximately 110°C.

Figure 6 represents the result of the differential scanning calorimetry investigation of the G4LZI3 protein. The thermogram shows a distinct, sharp peak, which is extrapolated to a temperature of 110°C. This temperature, referred to as the Tm of a protein, is an index of thermostability. Usually, the larger the Tm, the more thermodynamically stable the protein. Typically, proteins with higher Tm are less susceptible to unfolding and denaturation at lower temperatures.^22^ This result shows that the G4LZI3 protein may remain stable at temperatures lower than 110°C and turn out to be irreversibly denatured at higher temperatures above 110°C.

### Fluorescence spectrum of the G4LZI3 protein

**Figure 7:**
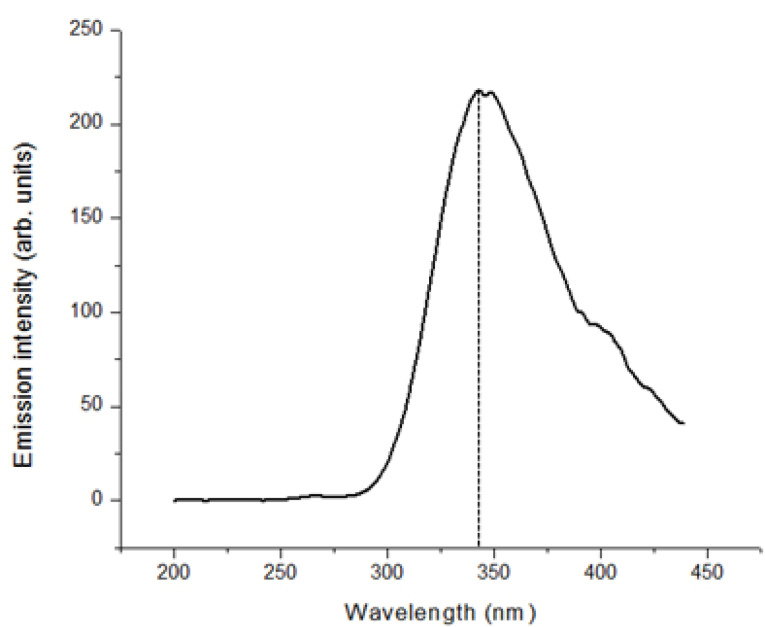
** Fluorescence spectroscopy spectra of G4LZI3.** The absorbance at approximately 340 nm is due to the fluorophores in the protein.

Fluorescence spectroscopy was carried out to study the alterations in the stability of the protein by detecting the intrinsic maximum fluorescence emission intensity of the fluorophores occurring in the protein. The result obtained, represented by the fluorescence spectrum in Figure 7, indicates that excitation at a wavelength of 270 nm produced a maximum fluorescence emission intensity of approximately 344 nm. This emission spectrum obtained is typical of the aromatic amino acid residues, predominantly tryptophan, phenylalanine and tyrosine, therefore indicating the stability and folded state of the USP.^23^

## Discussion

To the best of our knowledge, this is the first report of the successful heterologous expression, purification and preliminary biophysical characterization of the universal stress G4LZI3 protein from the *S. mansoni*parasite. The ability to express and purify a recombinant protein successfully is exceedingly vital for structural elucidation, biochemical characterization as well as functional studies on a protein of interest. To accomplish the objective of producing a considerable amount of recombinant *S. mansoni* G4LZI3 protein, an expression plasmid, pQE30-G4LZI3, was constructed and cloned. The codon-optimised pQE30-G4LZI3 plasmid was cloned into BamHI and HindIII cloning sites. It is important to mention that codon optimisation was done with the goal of increasing soluble recombinant G4LZI3 protein production by improving the translational efficiency during protein expression.^24,25^

Our study shows that the *E. coli*expression system is ideal for the production of the *S. mansoni* USP. Earlier studies had also reported the successful heterologous expression of USPs from numerous species of organisms, such as *E. coli*, *Salicornia brachiata* and *Mycobacterium*,using the *E. coli* expression system.^26-28^ SDS-PAGE analysis revealed that the recombinant G4LZI3 protein was present in the supernatant and not in the bacteria pellet, thus indicating that the protein was expressed as soluble protein rather than as insoluble aggregates, which suggests that overexpression of G4LZI3 with 0.5 mM IPTG and incubation at a temperature of 25°C overnight may be the ideal conditions for accomplishing expression of soluble G4LZI3 protein.

The clear soluble recombinant protein lysate was initially purified with IMAC using the AKTA FPLC purifier system. Two distinct bands corresponding to our protein of interest (20.3 kDa) and another protein suspected to be either a product of proteolysis or a toxin were also obtained, hence SEC was done as the second purification step to purify the G4LZI3 protein to homogeneity. Multiple-step chromatographic protocols in the purification of proteins meant for functional and structural studies is very essential to ensure about 99% purity of recombinant proteins.^16^

Previous reports indicated that proteins with histidine tags have very high affinity in IMAC owing to the presence of multiple histidine residues and are typically the strongest binders among other proteins present in a crude extract.^29,30^ The histidine tag was not cleaved from the protein after purification because small-sized fusion tags such as histidine tag, FLAG-tag, calmodulin-binding peptide tag and Strep-tag generally do not need to be cleaved prior to downstream applications, since they have a trivial influence on the physical characteristics and biological activity of the target protein.^31,32^ The outcome of our purification procedure suggests that affinity chromatography, followed by SEC, is ideal for the purification of recombinant G4LZI3 protein. This result is in line with the study of Jung and co-workers, which also reported successful purification of USP from *Arabidopsis thaliana*, AtUSP, using the affinity column.^33^

Subsequent to the production of purified recombinant protein, an important step is to carry out the preliminary biophysical characterization of the protein as per its functional group composition, secondary structure composition, folding state, thermal stability and purity. Characterization of a protein of interest can be achieved by using its structural, spectroscopic, electromagnetic, thermodynamic and biochemical properties, which are conferred on the protein owing to its composition. Production of proteins and subsequent structural and functional characterization exploit accumulative information from their fundamental general makeup (primary and secondary elements) and the diversity allowed by such composition.^34^ Biophysical characterization of a protein of interest is a necessary prelude to further structural characterization, and it helps in downstream therapeutic application. The production of soluble recombinant proteins appropriate for nuclear magnetic resonance (NMR) studies or X-ray crystallisation has been a key element in structural genomics programmes.^35^ The investigation of the secondary and tertiary structure of proteins is a valued process in quality control to confirm protein folding. Some of the techniques employed for this investigation include FTIR spectroscopy, fluorescence spectroscopy, ultraviolet spectroscopy and circular dichroism. In addition, stability studies of a protein are equally vital before structural characterization or determination, as these entail the assessment of a protein vulnerability to denaturation or degrading agents such as high temperature or oxidants.^36^

FTIR spectroscopy provides data on protein secondary structure content, which involves regular polypeptide chain arrangements of helices, sheets, coils, and turns that add up to form the tertiary structure. It is worth noting that FTIR spectroscopy is a useful tool for characterising protein secondary structure, even though this can also be established by circular dichroism spectroscopy. However, FTIR does not provide as much information as NMR and X-ray diffraction crystallography.^37^ FTIR spectroscopy involves the beaming of infrared radiation on a protein sample. Different compounds have a characteristic set of absorption bands in the infrared spectrum. Distinctive bands seen in the infrared spectra of proteins and polypeptides are the amide I and amide II bands, which arise from the amide bonds attached to the amino acids. The absorption related to the amide I band causes stretching vibrations of the C = O bond of the amide, while absorption linked to the amide II band leads mainly to bending vibrations of the N-H bond.^38^ Although FTIR spectroscopy is capable of providing valuable data about protein structure and the alignment of secondary structure, it cannot divulge information about protein mechanisms. Hence, the structural variations of each molecular group must be investigated.^39^ Results obtained from the characterization of the G4LZI3 protein using FTIR indicated that the protein contains predominantly the α-helix in its secondary structure. Earlier studies reported the effective usage of FTIR in the characterization of recombinant protein secondary structure elements.^40,41^ It is worth noting that the most subtle spectral region of a protein secondary structural component is the amide I band, which is usually within 1700−1600 cm^−1^; it is a result of the C = O stretch vibrations of the peptide linkages, which make up approximately 80% of a protein. The frequencies of the amide I band constituents are established to be correlated meticulously to every secondary structural element of a protein.^42^

Furthermore, the structural stability of the recombinant universal stress G4LZI3 protein was evaluated with differential scanning calorimetry and the result revealed that the protein is thermodynamically stable at temperatures below 110°C. Thermostability is a key factor to consider when producing recombinant proteins for subsequent downstream applications. A stable and well-folded protein is an important prerequisite for successful biotherapeutic application. More so, every biological process depends on proteins that are stable and properly folded.^43,44^

Fluorescence spectroscopy is an analytical technique appropriate for the analysis of the three-dimensional structure of proteins. The side chains of the aromatic amino acid residues, namely phenylalanine, tyrosine and tryptophan, exhibit fluorescence once they are excited. The exhibited fluorescence is usually dominated by the tryptophan side chains. This is because these have stronger absorption and intensity of emission than tyrosine and phenylalanine. There is usually a change in the fluorescence emission spectrum of a protein once there is an alteration in the structural conformation.^23^ Various studies have effectively used fluorescence spectroscopy in characterization and monitoring of the quality of recombinant proteins. This includes the detection and analysis of protein aggregation.^45-47^

Production of recombinant proteins and subsequent structural and functional characterization exploit cumulative information from their fundamental general make-up (primary and secondary structure elements) and the diversity allowed by such composition.^32^ Key determinants for further structural characterization and/or determination, and the usage of recombinant proteins as druggable targets or vaccine targets include foldedness, stability and solubility. Taken together, the outcome of this study is an indication that universal stress G4LZI3 protein is a promising protein target for drug discovery and development against schistosomiasis.

## Conclusion

This study reports the successful recombinant expression and efficient purification of the universal stress G4LZI3 protein from *S. mansoni.*Histidine-tagged G4LZI3 was expressed as soluble protein and was purified to homogeneity using both affinity and a size-exclusion purification system. Preliminary biophysical characterization of purified G4LZI3 showed that the protein is stable and will not be denatured during further structural characterization. Valuable information on the characteristics of the protein regarding its functional group composition, secondary structure composition, purity and thermal stability that are presented is crucial for further structural study to determine the protein mechanism of action in stress response and its application in disease intervention such as drug discovery or even vaccine development.

## Acknowledgments

The authors are appreciative of the University of Zululand for supporting the execution of this research.

## Ethical Issues

All experiments and procedures were carried out in compliance with the ethical principles of the University of Zululand and were approved by the university’s research and ethical committee.

## Conflict of Interest

The authors declare no conflict of interest regarding this study.
